# Revolutionizing diffuse uterine leiomyomatosis treatment: A case report and literature review on “no‐distension” hysteroscopic myomectomy with thoracic tissue forceps

**DOI:** 10.1002/ijgo.15800

**Published:** 2024-07-28

**Authors:** Zhengping Zhang, Haikun Yang, Ru Pan

**Affiliations:** ^1^ Department of Gynecology Oncologic Center Meizhou People's Hospital Meizhou China

**Keywords:** case report, diffuse uterine leiomyomatosis, fertility preservation, hysteroscopic surgery, non‐distension technique in hysteroscopy

## Abstract

Diffuse uterine leiomyomatosis (DUL) is a prevalent leiomyoma variant in women of childbearing age, characterized by a uniformly enlarged uterus with numerous interconnected small myomas. Given that most DUL patients are in their reproductive years, treatments that preserve fertility are increasingly vital. This case report introduces an innovative hysteroscopic technique that forgoes uterine distension to remove multiple submucosal fibroids in a single procedure, maintaining endometrial integrity and fertility. A 27‐year‐old single woman experienced prolonged and heavier menstruation. Magnetic resonance imaging (MRI) scans showed an enlarged uterus with several round‐like masses in the uterine wall/submucosa. Addressing the patient's financial limitations and treatment preferences, a groundbreaking hysteroscopic surgery was performed using thoracic tissue forceps, alongside bedside ultrasonography, enabling fibroid excision without uterine distension. In total, 38 uterine fibroids were successfully excised without complications such as uterine perforation or hyponatremia. According to the FIGO classification system: three were type III, nine were type II, 15 were type I, and 11 were type 0. Postoperative follow‐up indicated normalized menstrual cycles, improved hemoglobin levels, and no recurrence of fibroids. A hysteroscopic examination 1 month after surgery revealed no significant fibroids or endometrial thickening. This case report underscores the effectiveness of a novel hysteroscopic surgical approach in treating DUL. This method eliminates the need for multiple staged surgeries and the risks of endometrial damage inherent in traditional techniques. It offers a minimally invasive, fertility‐preserving alternative for young DUL patients, marking a significant advancement in gynecologic surgery.

## Synopsis

Innovative “no‐distension” hysteroscopic myomectomy with thoracic tissue forceps successfully treats diffuse uterine leiomyomatosis, preserving fertility and reducing complications.

## 1 INTRODUCTION

Diffuse uterine leiomyomatosis (DUL) is a relatively uncommon variant of leiomyoma, predominantly observed in women of childbearing age. It is characterized by a uniformly enlarged uterus with many small, interconnected myomas, typically each less than 3 cm in diameter.^1^ These myomas extensively infiltrate all layers of the endometrium and myometrium. Within the uterine environment, the presence of myomas is often associated with menorrhagia and infertility, significantly contributing to enlargement of the uterine cavity.^2^ In more severe cases of DUL, it becomes increasingly challenging to identify healthy tissue areas that are free from myoma infiltration.^3^ In managing DUL, hysterectomy is a common approach for patients not prioritizing fertility preservation. However, considering most DUL patients are of reproductive age, fertility‐conserving methods are becoming more crucial. Traditional myomectomy, although addressing multiple myomas, poses risks of damaging healthy tissue and potential pregnancy complications, presenting challenges in effective treatment.^4^ Advances in hysteroscopy and vascular interventions have led to the development of less invasive treatments for DUL, offering alternatives to more radical hysterectomy.^5^, ^6^ Recent studies, including those by Yen et al. and Zhao et al., highlight the success of hysteroscopic myomectomy and hysteroscopy endo‐operative system (HEOS) hysteroscopic surgery in preserving fertility and uterine health while minimizing surgical risks.^7^, ^8^ However, these methods often require fluid hysteroscopic distension and electrosurgical devices, posing risks such as intrauterine adhesions, uterine perforation, and fluid overload.^9^ Additionally, treating densely distributed fibroids may necessitate multiple surgeries, increasing patients' financial burden. Confronting these challenges in DUL treatment, particularly in fertility preservation and reducing surgical risks, our case explores an alternative therapy. We report on a 27‐year‐old DUL patient with dense submucosal fibroids, for whom multiple staged hysteroscopic surgeries were not viable. Therefore, we utilized a minimally invasive technique with thoracic tissue forceps, guided by hysteroscopy combined with ultrasonography, effectively excising all submucosal fibroids, and avoiding electrosurgical devices. This method minimized endometrial trauma and risks of fluid overload and uterine perforation. With ultrasound guidance, in a no‐distension uterine state, it offered a less impactful, single‐session surgical solution, bringing new hope to DUL patients.

## 2 CASE REPORT

**FIGURE 1:**
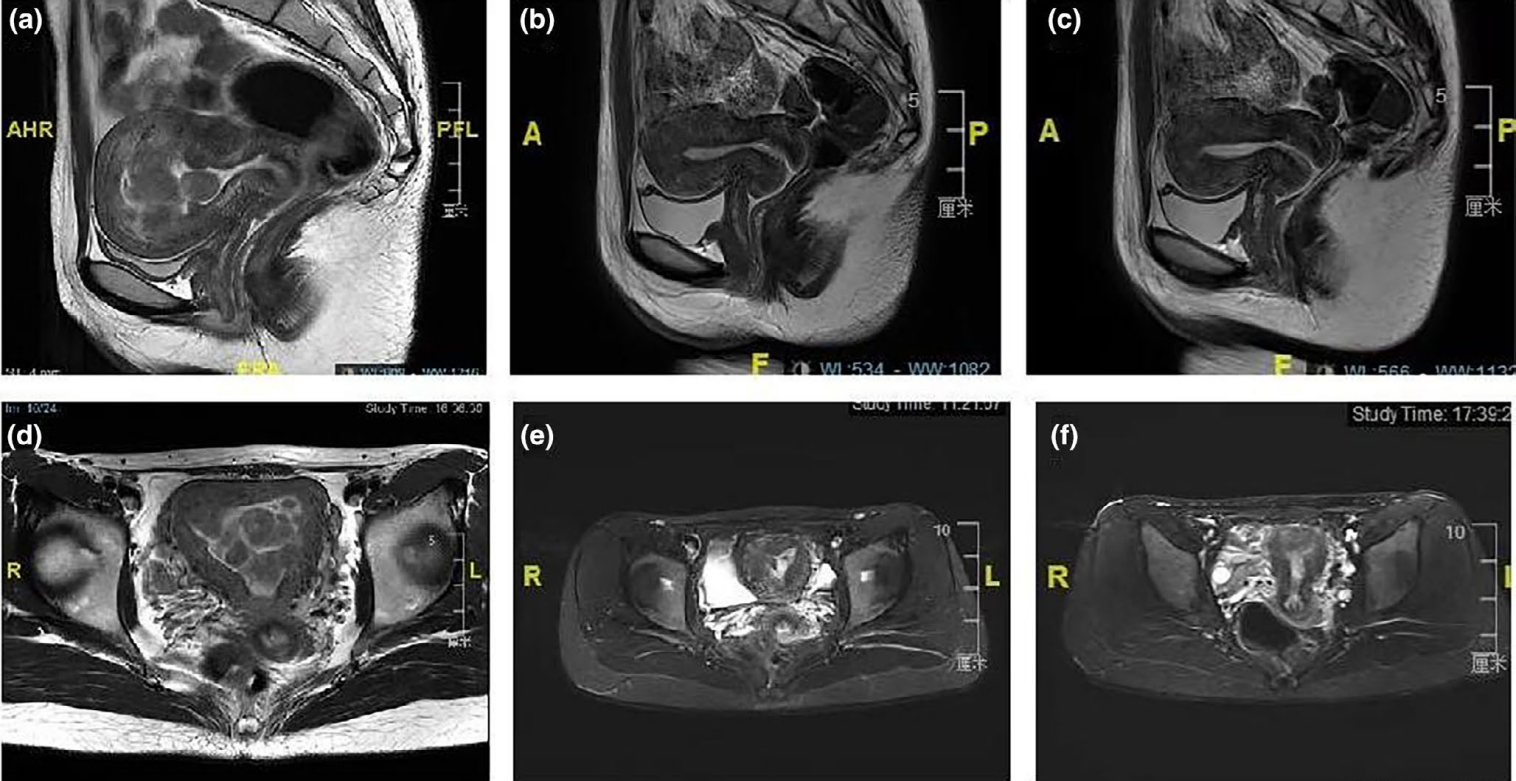
Pre‐ and postoperative pelvic magnetic resonance imaging (MRI) T2‐weighted images. (a) Preoperative sagittal view showing diffusely distributed submucosal leiomyomas in the uterine cavity. (b) One‐month postoperative sagittal view. (c) One‐year postoperative sagittal view. (d) Preoperative coronal view. (e) One‐month postoperative coronal view. (f) One‐year postoperative coronal view.

**FIGURE 2:**
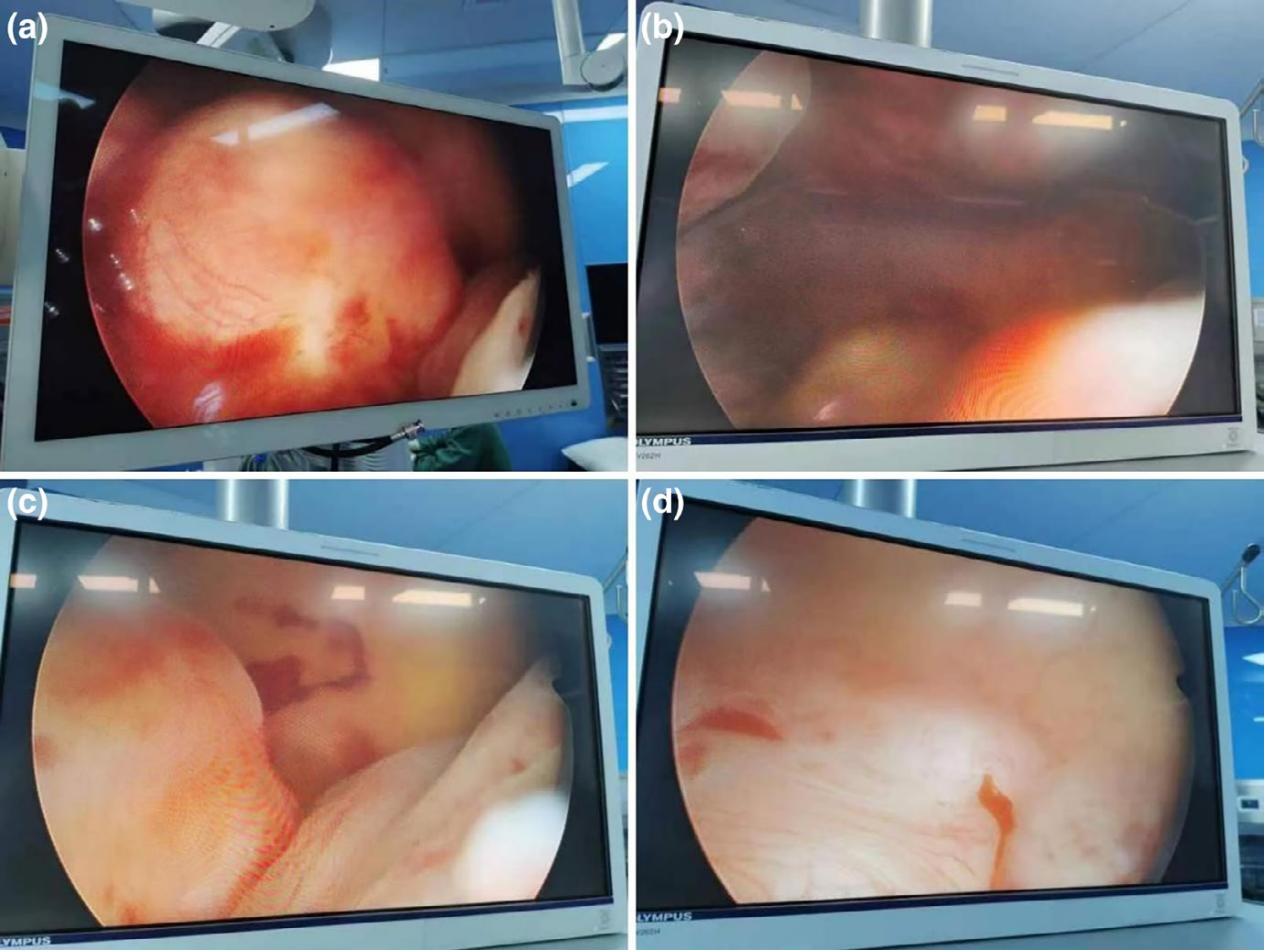
Hysteroscopic examination images. (a) Large submucosal fibroid protruding into the uterine cavity. (b) and (c) Narrowed uterine cavity with widespread fibroids. (d) Smaller submucosal fibroid located at the uterine fundus.

**FIGURE 3:**
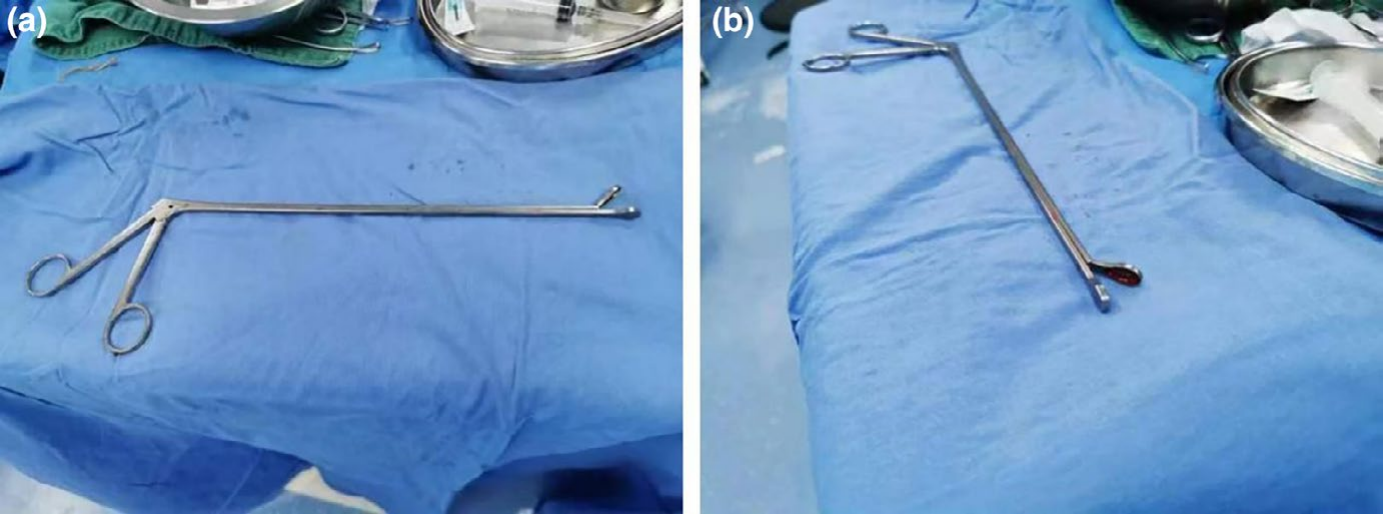
Thoracic tissue forceps. (a) Overall form of the tissue forceps. (b) The head of the forceps is round and blunt to minimize damage to the surrounding tissues, yet the contact surface of the forceps' tip is relatively sharp, enabling effective removal of the tumor while preserving the integrity of the surrounding tissues.

**FIGURE 4:**
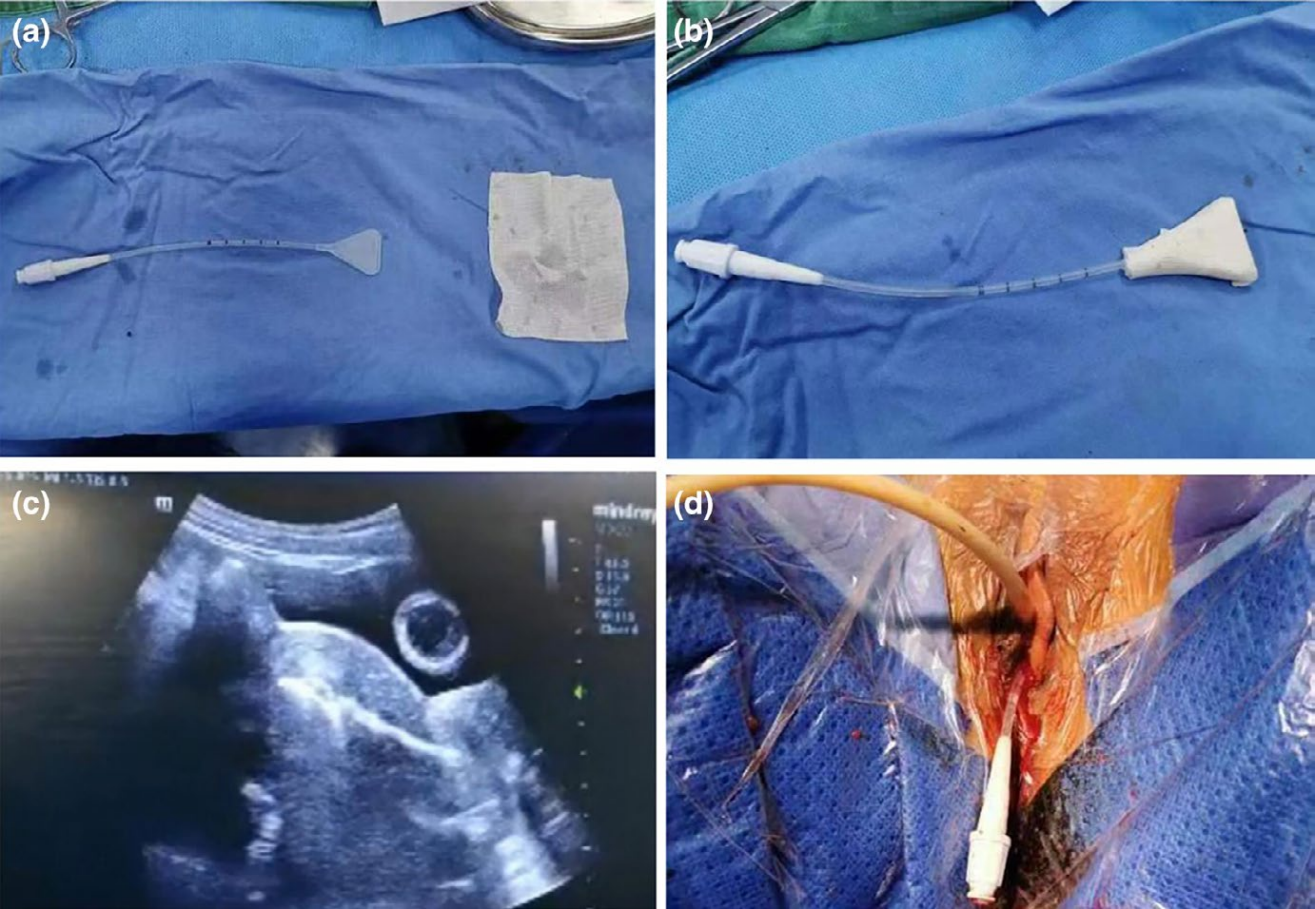
Placement of the hysteroscopic balloon. (a) Hysteroscopic balloon stent and adhesion barrier placement. (b) Adhesion barrier wrapped around the hysteroscopic balloon. (c) Placement of the hysteroscopic balloon stent under color ultrasound guidance. (d) Injection of 5 mL saline into the hysteroscopic balloon.

**FIGURE 5:**
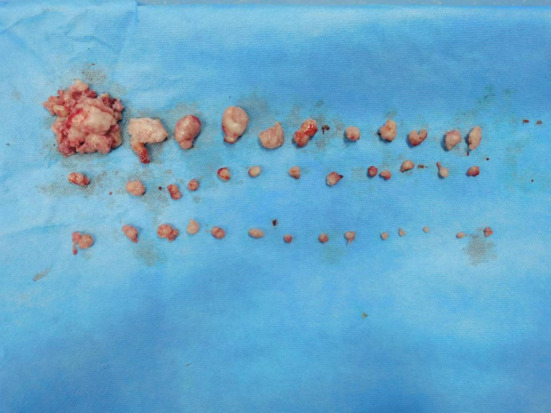
Total removal of 38 submucosal uterine leiomyomas.

**FIGURE 6:**
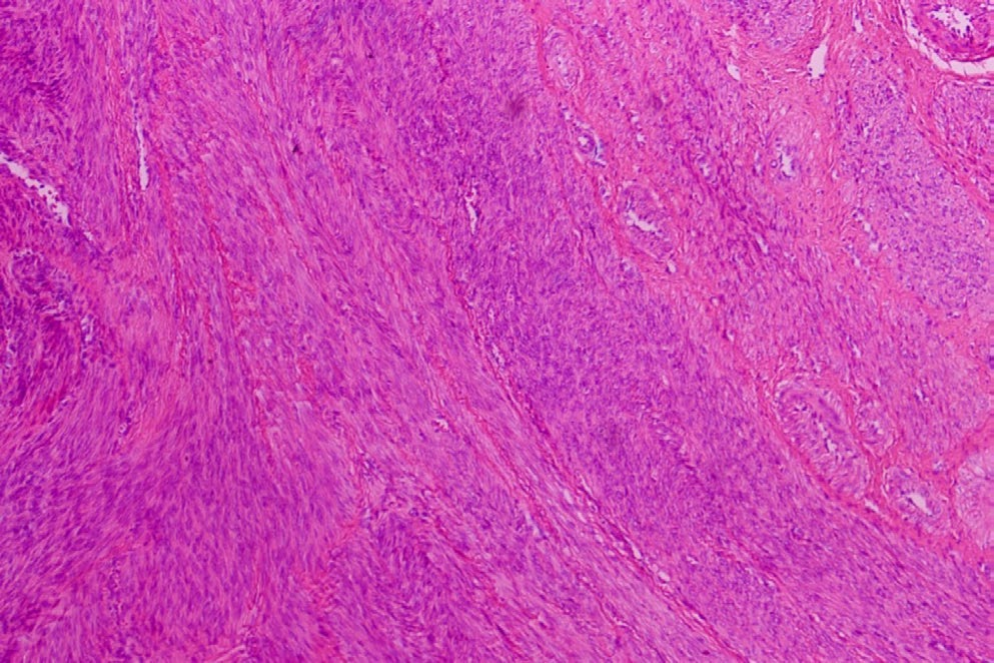
The lesion consisted of spindle‐shaped smooth muscle cells in a woven pattern, with unclear borders, eosinophilic cytoplasm, cigar‐like nuclei, and small nucleoli. Mitotic activity was low, as shown by hematoxylin & eosin (HE) staining.

**FIGURE 7:**
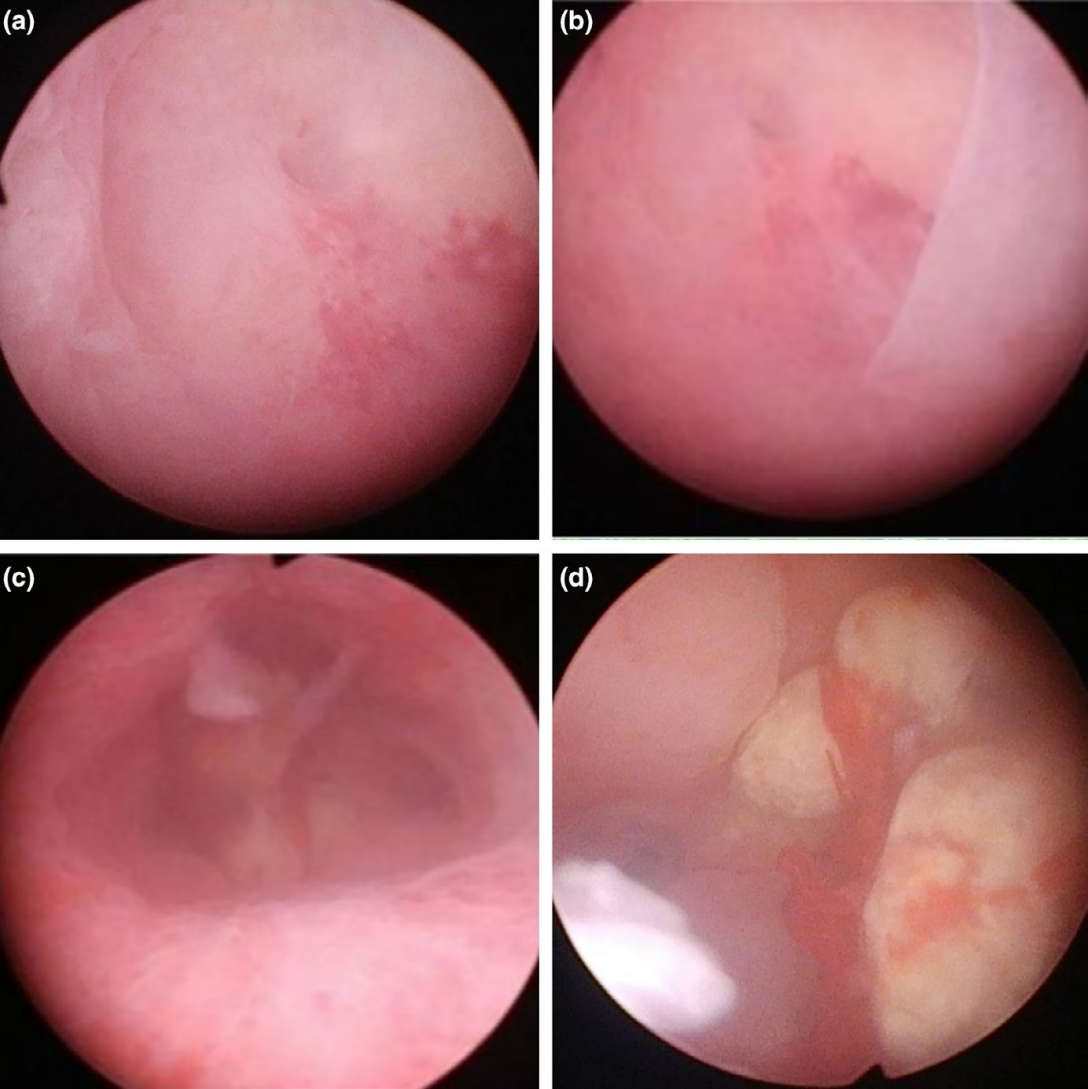
Hysteroscopic examination images. (a) Right uterine horn. (b) Left uterine horn. (c) Overall morphology of the uterine cavity. (d) Yellow flocculent material inside the uterine cavity, representing residual anti‐adhesion film.

This study was approved by ethic committee of Meizhou People's Hospital (2022‐C‐138). In this study, informed consent was obtained from the patient. The consent was obtained on October 7, 2022, through a written form signed by the patient. Before signing, the patient received a detailed explanation of the study's purpose, procedures, potential risks, and benefits, as well as assurances regarding the protection of their personal information and privacy. It was emphasized that consent was voluntary, with the right to withdraw at any time without adverse consequences. Additionally, all personally identifiable information has been de‐identified to safeguard the patient's privacy. A 27‐year‐old unmarried woman with a history of sexual activity visited our hospital due to prolonged menstrual periods (extending from the usual 4–5 days to 10–12 days) and increased menstrual flow (accompanied by large blood clots) over the past 4 months. She experienced menarche at 14 and currently has a menstrual cycle of 24–26 days, with no significant changes in the cycle pattern. Occasionally, she reported dizziness and fatigue but no post‐coital bleeding, abdominal pain, nausea, or vomiting. Gastrointestinal and urinary system examinations showed no abnormalities, and her mental state, sleep, appetite, bowel movements, and weight remained stable. She had no history of drug allergies and no significant medical, surgical, or family history. A gynecologic examination revealed an enlarged uterus, comparable in size to an 8‐week pregnancy. Auxiliary examinations included complete blood count, liver and kidney function tests, electrolytes, ECG, chest X‐ray, cardiac ultrasound, and ultrasounds of the liver and urinary system. The complete blood count indicated moderate anemia with a hemoglobin (Hgb) level of only 74 g/L. Liver and kidney function tests and electrolyte levels were normal. ECG, chest X‐ray, and ultrasounds of the heart and urinary system showed no significant abnormalities. Magnetic resonance imaging (MRI) revealed an enlarged, anteriorly positioned uterus with multiple round‐like masses between the muscle walls/submucosa, the largest being approximately 2.7 × 2.2 × 2.2 cm. These masses appeared isointense on T1‐weighted images, iso to slightly hyperintense on T2‐weighted images, slightly hyperintense on diffusion‐weighted imaging (DWI) sequences, with reduced ADC values. The enhanced scan showed uneven, marked enhancement, with patchy weak/non‐enhanced areas within some lesions. The uterine cavity was enlarged, showing patchy high and low signals on T2WI, with no clear enhancement on the enhanced scan (Figure 1a,d). Considering these findings, we diagnosed DUL with moderate anemia. In the preoperative assessment, we used the size, topography, extension of the base, penetration and lateral wall position (STEPW) scoring system, assigning eight points (Table 1). We recommended gonadotropin‐releasing hormone agonist (GnRHa) treatment to correct anemia and reduce tumor size, followed by surgical treatment, and informed her of the potential need for multiple hysteroscopic surgeries. However, the patient was unable to accept a long, multistage surgical treatment plan. Considering her unique situation, we organized a multidisciplinary discussion. As a young woman with predominantly submucosal fibroids, the use of laparotomy or laparoscopy might damage her endometrium, leading to pelvic adhesions, increased infertility risk, or ectopic pregnancy. If traditional hysteroscopic electrosurgery is employed, it may lead to extensive damage to the endometrium, with a high risk of postoperative intrauterine adhesions. Furthermore, due to the numerous fibroids, there is a risk of hyponatremia during traditional hysteroscopic surgery, which may require multiple procedures to complete, increasing the economic burden on the patient and causing additional damage to the endometrium. Therefore, we devised a personalized treatment plan for the patient, aimed at treating multiple submucosal fibroids in a single session while maximally preserving her fertility. This approach primarily involves the use of hysteroscopy combined with ultrasound‐guided clamping and excision of submucosal uterine fibroids. For the first time, we used thoracic tissue forceps (model HJH010708, Hangzhou Kangsheng Medical Equipment Co., Ltd., Hangzhou, China) as cold instruments (Figure 2) to excise submucosal uterine fibroids. The forceps' rounded, blunt head minimizes damage to surrounding tissues. Additionally, its gripping arm is equipped with sharp cutting blades, which effectively excise lymphoid tissue while preserving the integrity of the surrounding tissues. With these forceps, assisted by hysteroscopy and ultrasound, we could precisely excise submucosal fibroids. The surgical procedure is detailed in Video S1. A urinary catheter was inserted, and the uterus was probed to a depth of about 9 cm, revealing a patent cervical canal and a pear‐shaped uterine cavity with slightly thickened endometrium. Multiple uterine fibroids were observed within the cavity, some with rich surface vasculature, ranging in size from ~ 0.5 to 2–3 cm (Figure 3). Under ultrasound guidance, biopsy forceps were used to grasp and fix the part of the submucosal fibroid protruding into the uterine cavity, penetrating its capsule to reach the tumor body. By rotating and pulling, we disrupted the capsule and separated part of the tumor, which then protruded into the cavity. The remaining tumor was gradually clamped and excised under ultrasound monitoring. The final hysteroscopic review showed no tumors in the cervical canal or uterine cavity, and the endometrium was not thickened. After these procedures, an Interceed absorbable adhesion barrier (model 4530, Johnson & Johnson, USA) was wrapped around the uterine balloon stent (model ZJ‐5, Jiangxi Xinnuo Bioengineering Co., Ltd., China) and inserted into the uterine cavity under ultrasound guidance, followed by the injection of 5 mL of saline to reduce bleeding and prevent adhesion (Figure 4). The entire surgery lasted 50 min (15 min for hysteroscopic examination, 30 min for fibroid surgery in a no‐distension state, and 5 min for placing the uterine balloon stent), with 2900 mL of irrigation used and 2800 mL returned, and an estimated blood loss of about 20 mL. A total of 38 uterine fibroids were successfully excised (Figure 5). According to the FIGO classification system: three (7.89%) were type III, nine (23.68%) were type II, 15 (39.47%) were type I, and 11 (28.96%) were type 0. Postoperative pathology suggested typical uterine leiomyomas (Figure 6). No complications such as uterine perforation or hyponatremia occurred during or after the surgery, and the patient was discharged smoothly on the second day post‐operation. Ten days post‐surgery, the uterine balloon was removed. A one‐month follow‐up revealed a good recovery, with a complete blood count showing Hgb 109 g/L and MRI showing no recurrence of uterine fibroids (Figure 1b,e). A second office hysteroscopic examination also found no significant tumors. The uterine cavity was normal in shape, with no endometrial thickening. At the uterine fundus, we discovered a yellowish mass‐like substance, presumed to be residual anti‐adhesion film, which was scraped out (Figure 7). The patient resumed her menstrual cycle 40 days after surgery, with a cycle of 25–26 days, a period of 5–6 days, and normal menstrual flow without dysmenorrhea. A one‐year postoperative review showed Hgb 116 g/L and MRI revealed no recurrence of uterine fibroids (Figure 1c,f).

**TABLE 1:** Preoperative assessment utilizing the STEPW scoring system.

Points	Size	Topography	Extension of the base	Penetration	Lateral wall	Total
0	<2 cm	Low	<1/3	0	+1	
1	2–5 cm	Middle	1/3–2/3	<50%		
2	>5 cm	Upper	>2/3	>50%		
Score	1	2	2	2	1	8
0–4	Group I	Low complexity HM				
5–6	Score 5–6	High complexity HM, two‐step HM, GnRH agonist use				
7–9	Score 7–9	An alternative to HM to be considered				

Abbreviations: GnRH, gonadotropin‐releasing hormone; HM, hysteroscopic myomectomy; LMs, leiomyomas; STEPW, size, topography, extension of the base, penetration and lateral wall position.

The total cost of treatment during the hospital stay was ￥9150.95. If the patient had undergone the traditional myomectomy, it would generally require multiple surgeries, with each hospital stay costing around ￥11 000 to ￥12 000.

## 3 DISCUSSION

In the present study we report the innovative management of a case of DUL by using thoracic forceps hysteroscopically. After surgery, a total of 38 uterine fibroids, ranging in size from ~ 0.5 to 2–3 cm, were successfully excised. The total cost was decreased by more than a half, compared with traditional myomectomy. The one‐year postoperative visit showed that Hgb was 116 g/L and MRI revealed no recurrence of uterine fibroids.

DUL is characterized by widespread fibroids within the uterus.^1^ An MRI scan of a 27‐year‐old unmarried female revealed an enlarged uterus with multiple masses, consistent with DUL.^10^ The etiology may involve genetic factors,^11^, ^12^, ^13^ as indicated by the patient's symptoms and her mother's history of uterine fibroids.^14^

Hysteroscopic myomectomy is an effective, minimally invasive treatment for DUL,^15^ but traditional methods risk thermal injury and hyponatremia, especially with multiple submucosal fibroids. These risks can extend treatment duration and increase complications, affecting patient adherence.^7^, ^8^, ^9^, ^16^, ^17^

We used the STEPW system for patient assessment,^18^ scoring 8, indicating higher surgical complexity. Instead of pretreatment with GnRHa, we opted for a “no‐distension” hysteroscopic myomectomy using thoracic tissue forceps, combining hysteroscopic examination with ultrasound guidance. This approach limited distension time to 15 min with 100 mL fluid input, compared to longer times (35 min), higher fluid volumes (1000 mL) in traditional methods, necessitating the interruption of the surgery and planning for a secondary operation.^7^

Thoracic tissue forceps, typically used in thoracic surgery, allowed precise excision of fibroids while protecting surrounding tissues. We used the forceps to grasp and peel fibroids in a non‐distended state, avoiding the need for electrosurgical instruments. This strategy minimized endometrial damage and allowed complete fibroid removal, compared with the traditional hysteroscopic treatment methods.^8^, ^16^

In this case, after confirming the complete removal of fibroids using color Doppler ultrasound, we conducted a follow‐up hysteroscopic examination, which indicated neither significant bleeding nor residual fibroids. Most submucosal fibroids were successfully removed in a non‐distended uterine state, restoring the normal morphology of the uterine cavity. To minimize the risk of post‐surgery intrauterine adhesions, we placed the uterine balloon stent covered with Interceed absorbable adhesion barrier inside the uterine cavity. This approach aligns with the findings by Lin et al., who identified the uterine balloon as the most effective method for preventing adhesion reformation after hysteroscopic adhesiolysis in patients with Asherman's syndrome.^19^ Additionally, another study indicated that combining Interceed with estrogen therapy significantly reduced uterine adhesions and tissue fibrosis while improving endometrial receptivity.^20^ All these findings directly and indirectly support our decision to use the Interceed absorbable adhesion barrier, aiming to enhance uterine recovery post‐surgery and reduce the risk of infertility.

Furthermore, second‐look office hysteroscopy has proven to be a simple and effective method for prevention and treating intrauterine adhesions.^21^, ^22^ This technique allows for timely identification of mild to moderate adhesions and inspection of residual adhesion membranes or subtle adhesion bands. Therefore, 1‐month post‐surgery, we conducted a second‐look office hysteroscopy which revealed the good condition of the uterine cavity: the endometrium was smooth, uniformly textured and well‐vascularized. Moreover, the residual Interceed anti‐adhesion membrane was successfully removed. Postoperatively, to avoid the potential for hormones to promote fibroid recurrence, we did not use estrogen‐progestin therapy to facilitate endometrial repair. Follow‐up measurements showed normalization of the patient's menstrual cycle, improved hemoglobin levels, and no signs of fibroid recurrence. The patient expressed satisfaction with the whole treatment, further confirming the success of our surgical approach.

In the present study, we report the innovative management of a DUL patient by using thoracic forceps hysteroscopically, which efficiently excised the uterine fibroids in one single operation, decreased the risk of multiple surgeries, and halved the total medical costs. However, this study had several limitations. It was a single case from clinical practice. The success might not exclude the possibility by chance and cannot represent that it could be extended in other cases or other institutes, before further confirmation in a well‐designed large‐scale population study. Another limitation was that we were unable to follow‐up the fertility outcome, since currently the patient does not plan for pregnancy.

## 4 CONCLUSION

In conclusion, this case report suggested the efficacy of an innovative surgical strategy, combining “no‐distension” hysteroscopic myomectomy with thoracic tissue forceps. This strategy successfully mitigates risks associated with traditional hysteroscopic surgeries. The patient's normalized menstrual cycles and lack of fibroid recurrence highlight this method's potential as a superior alternative for managing DUL, especially in fibroid‐prone patients. This case underscores the importance of personalized, minimally invasive techniques in gynecologic surgery, offering valuable insights for future interventions.

## AUTHOR CONTRIBUTIONS

This study was conceptualized by Haikun Yang, while Ru Pan was responsible for gathering the data. The manuscript was composed by Zhengping Zhang, and the final document received contributions and approval from all participating authors.

## FUNDING INFORMATION

None.

## CONFLICT OF INTEREST STATEMENT

The authors have no conflicts of interest.

## Supporting information


**Video S1.** xxx.

## Data Availability

Data sharing is not applicable to this article as no new data were created or analyzed in this study.
