# Supplementary material for: Revolutionizing diffuse uterine leiomyomatosis treatment: A case report and literature review on “no‐distension” hysteroscopic myomectomy with thoracic tissue forceps
Source: Int J Gynaecol Obstet. 2024 Jul 28;168(1):87–93. doi: 10.1002/ijgo.15800 (PMC11649851; doi:10.1002/ijgo.15800)
Supplement: Supplementary file 1 — Video S1. xxx. [file IJGO-168-87-s001.zip › Supplementary video S1 video caption.doc]

## No-Distension Hysteroscopic Myomectomy Using Thoracic Tissue Forceps

### Description

This supplementary video provides a comprehensive demonstration of the no-distension hysteroscopic myomectomy procedure using Thoracic Tissue Forceps for the treatment of diffuse uterine leiomyomatosis (DUL). The patient is a 27-year-old woman who presented with prolonged menstrual periods and increased menstrual flow over the past four months, leading to a diagnosis of multiple submucosal uterine fibroids and moderate anemia.

### Procedure Overview

#### Case Presentation

• Patient Profile: A 27-year-old female presented with prolonged menstrual periods lasting 10-12 days and increased menstrual flow with large blood clots over the past four months.

• Menstrual History: Menarche at age 14, currently experiencing 24-26 day cycles. No significant changes in cycle pattern, occasional dizziness and fatigue, but no post-coital bleeding, abdominal pain, nausea, or vomiting.

• Diagnostic Tests: Hemoglobin level was 74 g/L indicating moderate anemia. MRI revealed an enlarged uterus with multiple intramural/submucosal fibroids, the largest measuring approximately 2.7 cm x 2.2 cm x 2.2 cm.

• Initial Diagnosis: Diffuse uterine leiomyomatosis (DUL) with moderate anemia.

#### Preoperative Assessment

• STEPW Score: The patient received a STEPW score of 8, indicating a high complexity myomectomy (refer to the STEPW Score table for detailed assessment criteria).

#### Surgical Procedure

• STEP 1: Preoperative Hysteroscopic Examination: A hysteroscope was used to visualize the uterine cavity, revealing multiple dense submucosal fibroids with some showing rich vascularization. The fibroids ranged in size from approximately 0.5 cm to 2-3 cm.

• STEP 2: Ultrasound-Guided Myomectomy with No-Distension Technique: Using ultrasound guidance, Thoracic Tissue Forceps (Model HJH010708) were employed to clamp and excise the submucosal fibroids. The technique involved grasping the fibroid, penetrating its capsule, and using a rotating and pulling motion to disrupt and remove the tumor. The forceps' rounded, blunt head minimized damage to surrounding tissues while the sharp cutting blades ensured effective excision.

• STEP 3: Postoperative Hysteroscopic Examination: After excision, a postoperative hysteroscopic examination was performed to ensure no residual fibroids remained.

• STEP 4: Placement of Uterine Balloon Under Ultrasound Guidance: An INTERCEED Absorbable Adhesion Barrier was wrapped around a Uterine Balloon Stent and inserted into the uterine cavity under ultrasound guidance. The balloon was inflated with saline to reduce bleeding and prevent adhesions.

#### Postoperative Follow-Up

• Menstrual Improvement: The postoperative menstrual cycle was reduced to 5-6 days with normal flow, no blood clots, and no dysmenorrhea.

• Hemoglobin Levels: Post-surgery hemoglobin levels improved to 106 g/L at 1 month and 116 g/L at 1 year.

• Second Office Hysteroscopic Examination: Confirmed a normal uterine cavity with no significant tumors or endometrial thickening.

• Pre and Post-Treatment MRI Comparison: Postoperative MRI showed a normal uterine morphology with no recurrence of fibroids.

This video provides a detailed visual guide to the no-distension hysteroscopic myomectomy procedure using Thoracic Tissue Forceps, demonstrating its effectiveness in treating diffuse uterine leiomyomatosis while preserving fertility and ensuring successful surgical outcomes.
